# Education and Our Educators

**Published:** 1912-12

**Authors:** Boris Sidis


### Education and Our Educators.

BY BORIS SIDIS, M. A., PH. D., M. D.

"Open the eyes of your children, that they may see," says Professor Sidis, father of Harvard's boy genius.

### "THE CHILD IS UNDER YOUR CONTROL."

Fathers and mothers, I assume that you are especially interested in the development of personality as a whole, the true aim of education.

I assume that you realize that what is requisite is not mere routine, not mere desiccated, quasi-scientific methods of educational psychology, not the sawdust of college-pseudogogics and philistine normal-school training, but more light on the problems of life. What you want is not the training of philistines, but the education of genius. The child is under your control.

We regard the child's mind as a tabula rasa, a vacant lot—and empty on it all our rubbish and refuse. We labor under the delusion that stories and fairy tales, myths and deceptions about life and man are good for the child's mind. Is it no wonder that on such a foundation men can put up only shacks and shanties? We forget the simple fact that what is harmful for the adult is still more harmful to the child.

Open the eyes of your children, that they may see.

### OUR SCHOOLS STERILIZE THE MINDS.

Our schools only sterilize, petrify and embalm their minds. They are pressed into one mold.

As in modern times our college authorities justify the brutalities of football and prize fights, so in ancient times the great moralists of those ages justified their gladiatorial games. The principle of recognition of evil under all its guises is at the basis of the true education of man. Is it not strange that this vital principle—a fundamental principle with the great thinkers of humanity—should remain so sadly neglected by our educators and public instructors?

### GENIUS SUPPRESSED.

The goody-goody schoolma'am, the mandarin schoolmaster, the philistine pedagogue, with his business capacities, have proved themselves incompetent to deal with the education of the young

They stifle talent, they stupefy the intellect, they paralyze the will, they suppress genius, they benumb the faculties of our children.

The educator, with his pseudo-scientific, pseudo-psychological pseudogogics, can bring up only a set of philistines with firm-set habits—marionettes, dolls. Teachers are blind leading the blind.

CHILDREN ARE CRUSHED UNDER THE INDUSTRIAL JUGGERNAUT.

Business is put above learning, administration above education, discipline and order above cultivation of genius and talent. Our schools and colleges are controlled by business men. The school boards, the boards of trustees of almost every school and college in the country consist mainly of manufacturers, storekeepers, tradesmen, bulls and bears of Wall Street and the marketplace. What wonder that they bring with them the methods of the factory, store, bank and saloon! The children are crushed under the industrial juggernaut.

AWAKEN CHILD'S LOVE OF KNOWLEDGE.

Awaken in early childhood the critical spirit of man; awaken early in the child's life love of knowledge, love of truth, of art and literature for their own sake, and you arouse man's genius.

It is claimed on good evidence that man possesses large stores of unused energy which the ordinary stimuli of life are not only unable to reach, but even tend to inhabit. Unusual combinations of circumstances, however; radical changes of environment, often unloose the inhibitions brought about by the habitual narrow range of man's interest and surroundings.

You have heard the psychologizing educator advise the formation of good, fixed, stable habits in early life. Now I want to warn you against the dangers of such unrestricted advice. Fixed adaptations, stable habits, tend to raise the thresholds of mental life and to inhabit the liberation, the output of reserve energy. Avoid routine.

There is a period in the child's life between the ages of 5 and 10 when he is very inquisitive, asking all kinds of questions. The inquisitiveness should by all means be encouraged and fostered.

INQUIRY AND GENIUS ARE MORE SACRED THAN ABSTRACT BELIEF, DOGMA OR CREED.

We should not arrest the child's questioning spirit, as we are often apt to do, but should strongly encourage the apparently meddlesome and troublesome searching and prying and scrutinizing of whatever interests the child. The spirit of inquiry, the genius of man, is more sacred than any abstract belief, dogma or creed.

EDUCATION SHOULD BEGIN BETWEEN THE SECOND AND THIRD YEAR.

In the large majority of children the beginning of education is between the second and third years. It is at that critical period that we have to seize the opportunity to guide the child's formative energies in the right channels.

You claim you are afraid to force the child's mind. This is an error. The same amount of mental energy used in those silly games which we think are specially adapted for the childish mind can be directed with lasting benefit to the development of his interests, intellectual activity and love of knowledge.

Being in a barbaric stage, we are afraid of thought. We are under the erroneous belief that thinking, study, causes nervousness and mental disorders.

LATER IN LIFE WE STUFF THEM LIKE GEESE.

We do not care to develop a love of knowledge in early life for fear of brain injury, and then when it is too late to acquire the interest we force them to study, and we feed them and stuff them like geese. What you often get is fatty degeneration of the mental liver.

The immunization of children, the development of resistance to mental germs, whether moral, immoral or religious, can be affected only by the medical man with a psychological and a psycho-pathological training.—From *Philistine and Genius*, published by Moffat, Yard & Co. and the American-Examiner.

---

### QUESTION BOX.

Don't forget that the Question Box is open to every reader of the "RED BACK" and that any question you may wish to ask will be answered by a specialist in that line. Just address your letter to the "RED BACK," 204 East Tenth street, Austin, Texas.

Will you tell me where I can obtain an article that will enlighten my boy on the perils of venereal diseases?—M. J.

The State Board of Health will send you, free of cost, a bulletin that contains the information you desire.

---

What is eugenics?—Teacher.

Eugenics is a study of the scientific propagation of the race—plainly, the breeding of human thoroughbreds.—M. D.

---

I am going to be married soon. Where can I get information that would be useful to me in view of this fact?—Young Man.

Subscribe for the "RED BACK" and also send to the National Genetic League, Washington, D. C., for a free copy of its pamphlet, "Facts for Bridegrooms."—F. F. B.

---

Is it natural for young girls to suffer cramps at the monthly periods?—A Girl.

No. A sense of fullness and heaviness in the pelvic regions is natural, but actual pain is abnormal. The cause should be sought for, as constipation, insufficient action of the kidneys, anemia and so on. The most common cause of "cramps" in young girls is due to an undeveloped condition of the female organs, which should be attended to by a physician.—Margaret Holiday, M. D.

---

Should a mother be alarmed if her young daughter is not sick regularly every twenty-eight days from the time she first matures?
—A Mother.

No. It is unusual for the regularity to be established from the beginning. No alarm should be felt unless the girl is anemic, run down and lifeless. It is often the case that three or six months will elapse between the first and second occurrence. Regularity should be established in about two years, and after being once established, should not become irregular, and if it does, the cause must be sought for and corrected.—Margaret Holiday, M. D.

